# Redesigning value-based hospital structures: a qualitative study on value-based health care in the Netherlands

**DOI:** 10.1186/s12913-022-08564-4

**Published:** 2022-09-22

**Authors:** Gijs Steinmann, K. Daniels, Fabio Mieris, Diana Delnoij, Hester van de Bovenkamp, Paul van der Nat

**Affiliations:** 1 Erasmus School of Health Policy and Management, Erasmus University Rotterdam, PO Box 1738, Rotterdam, 3000 DR The Netherlands; 2 Department of Value-Based Healthcare, St. Antonius Hospital, Nieuwegein, Utrecht, The Netherlands; 3 Scientific Center for Quality of Healthcare (IQ Healthcare), Radboud Institute for Health Sciences, Radboud University Medical Center, Nijmegen, The Netherlands; 4 National Health Care Institute (Zorginstituut Nederland), Diemen, the Netherlands

**Keywords:** Value-based health care, VBHC, Netherlands, Organizational design, Organizational structures, Value-based redesign, Care coordination

## Abstract

**Background:**

A crucial component of value-based health care concerns the redesign of organizational structures. In theory, hospital structures should follow value creation: addressing medical conditions for specific groups of patients over full cycles of care. In practice, however, it remains unclear how hospitals can reorganize themselves into value-based structures. The purpose of this study is to explore the ways in which Dutch hospitals are currently implementing and pursuing value-based redesign.

**Methods:**

This qualitative exploratory study used semi-structured interviews and a focus group for data collection. Transcripts were analyzed through deductive coding, for which we used Mintzberg’s theory on organizational structures, particularly his work on design parameters.

**Results:**

In their efforts to create more value-based structures, Dutch hospitals often employ a variety of liaison devices, such as project teams and committees. By contrast, the actual formation of units around medical conditions is much rarer. Outcome data are widely used within planning and control systems, and some hospitals partake in external benchmarking. Not all hospitals use cost indicators for monitoring performance.

**Conclusions:**

Value-based redesign is not necessarily a matter of radical changes or binary choices. Instead, as Dutch hospitals show, it can be an incremental process, with a variety of potential knobs to turn to various degrees. Health care executives, managers, and professionals thus have a wide range of options when they aim for more value-based structures. Our conceptualization of “value-based design parameters” can help guide the selection and implementation of strategies and mechanisms for further coordination around medical conditions over full cycles of care.

**Supplementary Information:**

The online version contains supplementary material available at 10.1186/s12913-022-08564-4.

## Background

The organizational structures of hospitals have repeatedly been criticized for impeding coordination, hampering efficiency, and delivering suboptimal patient care [1–3]. Moreover, much of this critique is supported empirically [2, 4–6]. In this regard, the recent and widespread uptake of value-based health care (VBHC) is of particular interest since a key component of VBHC concerns the redesign of organizational structures [3, 7, 8].

Although parallels exist between VBHC principles and approaches such as process-based design [2], VBHC distinguishes itself by the way it defines and emphasizes value. In health care delivery, the argument goes, value consists of what matters most to patients: the health status they achieve (outcomes) and the resources needed to reach that status (costs) [7]. By relating outcomes to costs, value encompasses efficiency and establishes an overarching aim for health care systems: *to optimize value by continuously striving to achieve the best outcomes as efficiently as possible* [7]. A foundational premise within VBHC—especially regarding organizational design—is that value is created at the level of medical conditions over full cycles of care ([7] p99-105). The idea is that value is not created at levels as broad as organizations such as hospitals or at levels as narrow as separate medical specialties or procedures, but over a full cycle of interdependent activities that together generate value for patients with a particular medical condition ([7] p44-51&203), [8].

For hardline VBHC proponents, a deep appreciation of this premise comes with three interrelated implications. The first is that providers should start to systematically measure both the outcomes and costs of their care cycles for each of the medical conditions they treat [3, 7, 8]. Second, provider organizations should realign their organizational structures with the goal of value and the level at which it is created. Thus, rather than organizing around medical specialties, hospitals ought to create integrated practice units (IPUs) that coordinate the full cycle of services necessary to treat patients with a particular medical condition ([7] p167-77). In our subsections below, we elaborate on this implication and the notion of value-based redesign. Third, the payment structures (i.e. procurement contracts) should also be in line with value creation: with bundled payments for full cycles or episodes of care for patients with a particular medical condition ([7] p265–67).

While the pioneering work on VBHC has informed a range of health policies across the globe [9–12], the actual reorganization into value-based hospital structures remains unclear and understudied [10, 13, 14]. This study aims to provide a deeper understanding of how hospitals realign their organizational structure with the creation of value for patients. Our research zooms in on the Netherlands, a country in which VBHC is high on the national health policy agenda and where multiple hospitals have started implementing VBHC principles [15, 16]. Therefore, we examine *how Dutch hospitals are currently working toward value-based redesign*: structural coordination around medical conditions over full cycles of care. Accordingly, we offer insight into the various ways in which value-based redesign is established in practice.

### The structuring of hospitals

In general, all organized activities require both a division of labor into specific tasks and the coordination of those tasks. An organizational structure, basically speaking, refers to the way in which task allocation and coordination are designed ([17] p2). Most of today’s hospitals are structured around medical specialties, with organizational units that are based on specific knowledge and skills (i.e. the functions) that are needed to perform certain complex tasks [1, 18]. Thus, hospitals typically have what is called a *functional design*: an organizational structure based on specialized skills [1, 17].

A main benefit of a functional design is that it facilitates contact and communication among similar (medical) specialists, thus supporting the continual transmission of complex skills [1, 17]. A downside, however, is that these structures are prone to pose workflow problems, resulting from a lack of coordination between organizational units [17]. This can become particularly problematic when the specialized activities within the various units are highly dependent on one another—such as in hospitals [1, 3]. Consequently, much of the criticism of hospitals’ functional design revolves around issues of interdependency and a lack of coordination between units [1–3, 18].

### Value-based redesign

Value-based redesign—task allocation and coordination around medical conditions over full cycles of care—would disrupt hospitals’ traditional structures [3, 7, 8]. According to the pioneering scholars on VBHC, such disruption is critical: improving value for patients requires a “fundamental restructuring” of the way health care delivery is organized [8].

A value-based approach will require challenging conventional wisdom and making changes in structures and practice patterns that have been in place for decades [8].

In practice, however, profound structural changes such as these are highly challenging, particularly in organizations such as hospitals, where a highly professionalized workforce operates within firmly established traditional structures [1, 19]. Additionally, most of the changes professed by hardline VBHC proponents are primarily described conceptually, and several scholars have expressed the need for a deeper connection with real-life organizational complexities, including more explicit guidance that can aid providers in their internal reorganization process [10, 20].

In this article, we build on Henry Mintzberg’s [17] research synthesis on the structuring of organizations, in which he elaborately addresses, among other topics, the mechanisms by which organizations arrange and coordinate their work and the reasoning behind them. Mintzberg is a renowned scholar in the field of management and organization studies, and his widely cited “*The Structuring of Organizations”* (1979) remains highly relevant today—something our current study re-emphasizes. Here, to examine *how* Dutch hospitals work toward value-based redesign, we particularly build on his conceptualization of “design parameters” ([17] p65-213). For Mintzberg ([17] p65), organizational design essentially comes down to “turning the knobs” that affect the division of labor and modify the mechanisms that coordinate work within an organization. In slightly more technical terms, these knobs are labeled the “design parameters” of organizational structures ([17] p65).

In Table 1, we list Mintzberg’s eight design parameters (first column); we describe the main ways in which each parameter can be used to organize the division and coordination of work (second column); and we outline our own conceptualization of “value-based design parameters” (third column), referring particularly to value-based redesign: task allocation and coordination around medical conditions over full cycles of care. The first and second columns are strictly based on Mintzberg’s compelling synthesis of research on organizational structures [17]; the third column is derived from our own synthesis of Mintzberg’s design parameters and Porter’s seminal texts on VBHC [7, 8]. Thus, our conceptualization of “value-based design parameters” refers to potential “knobs” that can be turned to modify the mechanisms that coordinate the interdependencies between the various people and activities involved in treating patients with a particular medical condition. See Additional File 1 for an elaboration on the theory from which Table 1 is derived.

**Table 1:** A synthesis of Mintzberg’s design parameters and Porter’s VBHC principles

Design parameter	Mode of coordination and division of tasks	Value-based design parameter
1. Unit size	Usually, units with more members will rely more on various forms of standardization for coordination; smaller units allow for more frequent and immediate interactions, and can thus more easily rely on mutual adjustment and interpersonal relationships	Value-based sizing refers to the process by which the size of organizational units is taken into consideration regarding coordination around medical conditions over full cycles of care
2. Unit grouping	By grouping positions (i.e. employees with certain roles and tasks) into units, an organization establishes its formal authority structure, which enables coordination through direct supervision. Additionally, grouping encourages frequent (informal) communication among unit members, thus facilitating coordination through mutual adjustment	Establishing value-based organizational units around medical conditions (instead of specialty-based departments)
3. Liaison devices	These devices facilitate mutual adjustment, mainly through informal communication, *between* units. They constitute contacts (liaisons), such as meetings and positions, that are woven into the formal structure to spur coordination across unit boundaries	Formally establishing contacts between units that are aimed at the coordination of activities around a medical condition (over the full cycle of care)
4. Planning and control systems	These systems generate the standardization of *output* (the results of work). Plans specify a desired standard; controls assess whether a standard is achieved	Utilizing outcome and cost measurements as value-based performance indicators
5. Training and indoctrination	Enabling coordination through the standardization of skills, norms, and specialized knowledge	Propagating information and knowledge concerning VBHC within the organization
6. Job specialization	Key parameter for the division of labor; enables the organization to match people to tasks, fostering specialization and efficiency	Task delegation specifically targeted at coordination around medical conditions
7. Formalization of behavior	Standardizes work processes through predetermined regulations; activities are tightly coordinated, thus formalizing workflow	Establishing value-based clinical pathways for groups of patients with a particular medical condition
8. Decentralization	Altering the way in which decision-making power is distributed within the organization. Decentralization refers to the dispersal of decision-making power	Value-based decentralization occurs when value-based units acquire more decision-making power

For the purpose of our study, “unit grouping” is a particularly relevant design parameter since the notion of an IPU can be regarded as the ideal type of organizational unit within VBHC theory. However, within this study, we distinguish these ideal type IPUs from what we conceive of as “value-based units.” In the context of hospitals, an IPU would acquire and manage its own budget and ideally be an independent “profit-and-loss center” [3]. Thus, next to shifting lines of authority, reorganizing into IPUs would break up the traditional flow of funds through specialty departments [3]. What we conceive of as value-based units, however, does not necessarily imply a shift in financial structures. Nevertheless, these value-based units are *formally grouped* together into distinctive parts of the organization (e.g. in a breast cancer department); they are assigned official authority within the hierarchy of a hospital. Accordingly, they differ from interunit multidisciplinary *teams*, which are informal parts of the organization (i.e. liaison devices that overlay the formal structure).

## Methods

To explore the ways in which Dutch hospitals are working toward more value-based structures, this qualitative study made use of semi-structured interviews and a focus group for data collection. Throughout the research, we have built on our synthesis of organizational design parameters [17] and VBHC [7].

For this study, the need for ethical approval was waived by the Medical Ethics Committee Erasmus MC (MEC-2019–0189).

### Setting

Our research focuses on the organizational structures of hospitals (outpatient specialty clinics do not fall within the scope of this study). In hospitals—relatively large health care organizations that provide a wide range of services out of a traditionally well-established functional structure—the organizational changes professed by hardline VBHC proponents seem particularly challenging.

The Netherlands forms an interesting setting, as the concept of VBHC is currently being adopted by a variety of organizations, including national policy institutions, health care insurers, hospitals and other provider organizations [15, 16]. The Dutch health care system is characterized by regulated or managed competition. Roughly speaking, insurers are encouraged to compete for members by offering attractive premiums, which should incentivize them to critically purchase health care provision, thereby stimulating providers to demonstrate quality and efficiency. A crucial piece of regulation concerns the mandatory health insurance package that each citizen is required to take on and each insurer must cover for any (potential) member (at an equal price irrespective of individual characteristics). This basic insurance package aims to ensure the accessibility and affordability of high-quality health care provision, covering family care, specialist care, and inpatient hospital care, among others [21].

In total, the Netherlands currently has 69 hospitals (including eight academic hospitals). Within the system of regulated competition, all of these hospitals are private not-for-profit organizations [22]. Academic hospitals are required to contractually employ their medical specialists (i.e. have them on payroll, similar to all nursing and most other staff). However, the majority of medical specialists working in general hospitals are not salaried employees but rather self-employed consultants within a closed hospital system. The contractual relation between consultants and the hospital is not arranged on an individual level but through a so-called “corporation” of medical specialists (*Medisch Specialistisch Bedrijf*). In essence, these corporations form within-hospital firms; they collectively negotiate contracts with a hospital, and fees are divided internally among members, usually differing between medical specialties [22].

### Sampling

Keeping our main objective in mind, we made use of purposive sampling [23], targeting hospitals that explicitly claim to be working toward value-based organizational structures. Therefore, we built on our professional network combined with gray and academic literature on VBHC in the Netherlands, which led us to list sixteen hospitals. Next, we contacted each hospital via e-mail. We briefly explained our research before asking 1) whether the respective hospital is indeed working on value-based organizational structures around medical conditions; and 2) if so, whether we could interview a suitable representative from within the organization. In most cases, our professional network allowed us to either directly contact a potentially suitable hospital representative or to contact a particular hospital employee in search of a referral; in other cases, we contacted the hospital’s main secretariat. Ultimately, we left it up to the potential interviewee to determine—based on the background information we provided about our research topic and objectives—whether he or she would be a suitable representative.

### Data collection

Between April and November 2020, we conducted a series of semi-structured interviews with representatives of Dutch hospitals. For this study, we composed an interview guide founded upon our theoretical framework (see Additional File 2). Hence, our questions focused on the ways in which hospitals are coordinating (or attempting to coordinate) health care delivery around medical conditions over full cycles of care.

We complemented our interviews with a focus group, for which we again made use of purposive sampling. Accordingly, we focused on the Linnean Initiative: an open multidisciplinary knowledge network that aims to accelerate the implementation of VBHC in the Netherlands [24]. One of their nine workgroups focuses specifically on the transition toward integrated practice units (IPUs). Within this IPU workgroup, “the frontrunners in the field of value-based care in the Netherlands are considering this issue and are developing a step-by-step plan to build towards an archetypal IPU” [25]. We organized an online focus group in which the members of this IPU workgroup would discuss our theoretical framework and our initial findings from the interviews, which we briefly presented beforehand (see Additional File 2 for our topic list). Through their hands-on expertise and their active involvement in an independent national knowledge network, the data from this focus group were used to strengthen our findings.

The interviews were conducted by either the first author (GS) or the third author (FM). At the time of the interviews, the first author was a male PhD candidate with an educational background in cultural anthropology, whose research focuses on VBHC in the Netherlands. The third author was a male student within the Health Sciences, Healthcare Policy and Management bachelor program (*Gezondheidswetenschappen, Beleid & Management Gezondheidszorg*) and was doing an internship at a VBHC department in a Dutch general hospital at the time of the study. Next to the first and third authors, the focus group was also attended by the second author (KD): a female PhD candidate with an educational background in health care policy and management, doing her research out of the same hospital department (VBHC) where the third author was doing his internship. Together, the first, second, and third authors conducted the data analysis.

### Analysis

Both the interviews and focus group were recorded and transcribed verbatim. All transcripts were analyzed through deductive coding [26]. We converted our theoretical framework into a coding scheme, in which the design parameters (see Table 1) formed the initial codes. Although we employed a predominantly theory-driven deductive coding process, we did remain sensitive to relevant findings that would not fit easily into the initial coding scheme [26]. See Additional File 3 for our final coding scheme.

The entire coding process, from the development of the initial coding scheme to the coding of all transcripts, was conducted by two primary coders (first and second authors). A third of the transcripts were coded in tandem, and the other two-thirds were coded individually by both coders, who discussed all conflicts and potential adaptations or additions to the coding scheme. Accordingly, we aimed to reduce variability within our analysis [27]. From October 2020 onward, all authors met regularly in group sessions to discuss the preliminary results and earlier drafts of this paper.

## Findings

Representatives of eleven hospitals agreed to partake in an interview (*n* = 11); three hospitals refused to partake due to COVID-19; one declared itself a poor match for our study; and another did not respond to our request. Of the eleven interview participants, nine represented a general hospital, and two represented an academic hospital. At the time of the interviews, four participants worked as a “Program Manager VBHC” and one as a “Project Lead VBHC”. An additional four worked on a hospital’s organizational strategy and innovation team: two as an “Advisor”, one as a “Project Manager”, and another as a “Program Director.” We also interviewed a “Chair Oncological Center” and a “Medical Director.” The interviews lasted 57 min on average.

Regarding the focus group, seven of the ten members of the Linnean Initiative’s IPU workgroup participated in a 90-min digital session. The focus group consisted of two hospital employees whom we interviewed before; two employees of hospitals from which we interviewed someone else; three health care consultants; and one delegate of a government institution.

Our synthesis of Mintzberg’s design parameters and Porter’s principles of VBHC—see Table 1 for an overview—formed the basis of our analysis and laid the groundwork for this section. Thus, each of the following subsections addresses a separate design parameter and how these are utilized by Dutch hospitals to coordinate work in line with the principles of VBHC. In our description, we adhere to Mintzberg’s [17] terminology (e.g. “unit grouping”, “standing committee”, “liaison devices”, “indoctrination”). When quoting respondents, we refer to them with a particularly assigned number in parentheses; expressions from the focus group are referred to by the number twelve (12).

### Unit size

In each of the interviews, the topic of unit size was brought up by the researcher. However, none of our respondents indicated that value-based sizing—the process by which the size of organizational units is taken into consideration in relation to enhanced coordination—was a particularly relevant item.

We have chosen to not express that in a number of millions or a number of employees, but rather just to check with common sense: what would be good homogenous groups [of patients] for which you can put together a [value-based] unit (7).

Thus far, the issue of size was relevant only in relation to the number of team leaders or the core set of team members who met and regularly discussed interunit affairs.

### Unit grouping

The concept of “value-based grouping” refers to the establishment of hospital units around medical conditions. Within Dutch hospitals, the considerations concerning the formation of units were relatively comparable. For instance, most interviewees expressed the belief that value-based units could indeed enable closer collaboration among everyone involved in treating patients with a particular medical condition. However, hospitals had acted upon this recognition in different ways.

I think you have two possible change strategies. One is that you have an idea, top-down, and you force it upon the organization, based on some kind of blueprint. [O]r, you let it arise organically from practice, bottom up, because the demand for a new organizational structure comes up. And that is the choice [our hospital] made (2).

Most hospitals opted for a more bottom-up approach in regard to value-based grouping. Accordingly, several hospitals had started “pilots” (1, 11,) in which they established multidisciplinary teams around a relatively small number of medical conditions. Respondents stated that the idea was to eventually create more of these teams and to incrementally carve these teams into the formal organizational structure.

Multidisciplinary, around a medical condition, we have now four [teams]. [E]ach of those [multidisciplinary teams] has a daily leadership board. [A]s the daily management of the team, the leadership board is responsible for the quality of care within such a team. [N]ow, we are mainly concerned with really working from within those multidisciplinary teams, that people know each other, know the process that a patient goes through, and know what the most important objectives are and shape that into a whole. [W]hat we are working toward is that these teams will be incorporated into the organizational structure (3).

But in small parts, of course. You could first start with those three integrated units with which we have started. So, a gradual transition (1).

Not all hospitals applied such an incremental approach. One, in particular, consciously made a different choice regarding the grouping into units around medical conditions:

We discussed that this was going to be the new reality, and that means that people have just switched from A to B. [T]hen, that also means that everyone around [those medical conditions], that those people are just added to another flow. So, we have discussed it and said: listen, we are going to organize it differently. [W]e made various patient flows, what we call [value-based units]. So, for example, the breast cancer flow contains the doctors, nurses, the breast cancer department. They are all added to this patient flow, and together they are responsible for finances and quality (7).

In sum, the respondents widely recognized that grouping into units around medical conditions could enable hospitals to better address the interdependencies of various activities that are needed to care for patients. Yet, most hospitals were hesitant to radically transform their traditionally functional unit structure into multidisciplinary value-based units around medical conditions.

### Liaison devices

Rather than switching to units around conditions, most hospitals were trying to increase coordination *between* their functional units through various types of liaison devices.

#### Liaison positions

To start, hospitals were making use of liaison positions. In fact, most hospitals had appointed a VBHC manager precisely to foster interunit coordination around medical conditions.

I work as a program manager VBHC. [S]o yes, in essence, I am responsible for setting up and continuing the VBHC program within our organization. So, the roll-out of care around medical conditions. And adding value, for the patient (1).

More specifically, these managers were assigned the task to coordinate the work conducted in several distinct units; they should have a “primarily supporting role” and “help teams organize themselves around a clinical pathway as best as possible” (11). Some respondents stated that in their hospitals, these managers are usually approached by a group of medical specialists, who then ask for support related to VBHC. In other organizations, it was usually the other way around, and managers had to actively search for potential cooperation.

I go to the highest manager below the executive board and ask “hey, on which medical conditions do you want to work in light of VBHC?” Because I need to know what is interesting for the hospital. Then, I go to that physician […] and we discuss the matter one-on-one. Afterward, we see who else we need to include, but it usually starts with me and a specialist. From there, I’ll work things out and discuss with the physician how to get things off the ground (8).

At the time of the interviews, some hospitals had a single VBHC manager who was operating relatively autonomously, but it was not uncommon for hospitals to have several managers with complementary roles in a VBHC management team. The exact composition of these VBHC management teams varied considerably among hospitals. In some organizations, the program manager was accompanied by a single medical specialist (a medical manager). Others had appointed a few more members, each focusing on a specific aspect of VBHC (e.g. one focusing on building a data infrastructure, one on work-process optimization, one on cost measurement) (10). Overall, the primary role of these VBHC managers was to foster communication between separate units that are involved in the full cycle of care for a medical condition. In practice, they were often doing this by utilizing another type of liaison device.

#### Standing committees

To facilitate mutual adjustment, hospitals had commonly established what Mintzberg ([17], p163) labels “standing committees,” referring to institutionalized meetings that take place regularly and enable interunit communication. In general, these committees are not temporal project teams but permanently woven into the official structure ([17], p163). So, in hospitals, “value-based” committees somewhat resemble value-based units in that they bring together a multidisciplinary group of employees around a medical condition. However, these committees are not official units; *they are liaisons*, overlaying the formal (functional) structure.

Within the project, we did not just look at the organizational structure but also at the meetings and consultation structure that goes with it, and we have set that up so that you can exchange and switch faster […] so, more of different levels, putting different disciplines together (6).

At the time of our data collection, developing these liaison “committees” had been *much more common and widespread than the actual formation of units* around a medical condition. In a common pattern, these committees started with a kickoff meeting, in which a large multidisciplinary group partook in determining the overarching mission and goal of the multidisciplinary interunit teamwork. After the kickoff, hospitals moved on to regular meetings—monthly was a common timeframe—to discuss their performance with a select group of delegates from the various specialties involved.

It is a periodic meeting in which basically the team gets together, [those] who are involved in the care of the medical condition. [A]nd in such a meeting, based on KPIs, they look at which outcomes can be improved. A nurse will also join, so basically everyone who should be involved, so someone from business intelligence also joins. And, yes, then you will basically determine “which KPI now requires the most attention, and which actions are we going to [undertake] to improve it?” (1).

Now, although these value-based committees are not actual units, in some hospitals, these committees did form the basis for the “pilots” in which the value-based units around a medical condition were being experimented with (see the section on Unit grouping above).

#### Matrix structure

As we have seen, at the time of our interviews, several Dutch hospitals were forming multidisciplinary units around a medical condition. However, this does not imply they were intending to sacrifice their functional units. Instead, they were conceiving of a transition toward a matrix structure—an organizational design that combines functional and value-based units.

We are trying to insert a kind of matrix structure. When you look at an organizational structure, then you’ll see the specialties on the vertical lines, and the care paths horizontally run through them. At this moment, the [hospital name] has chosen not to make the switch, and maybe we will never do that, because in the end it will be a matrix anyway. You want coordination within the specialties, but you also want coordination across specialties (9).

Not everyone appeared to be convinced, though, of the desirability of such a matrix structure. A recurring theme—regarding the matrix structure but also regarding value-based redesign in general—was that the end goal, the ideal structure, should be determined along the way.

A disadvantage of a matrix organization is that it will generate a lot of coordination at the intersections between vertical and horizontal management. If that is going to cause a lot of hassle, this can be a reason to eventually switch completely, in one direction or the other (3).

In sum, what applies to the (possible) transitions toward a matrix structure, in many cases applies to the use of all liaison devices: hospitals were utilizing them with caution, incrementally tweaking and experimenting with various types of connections between units.

### Planning and control systems

Value-based planning and control systems refer to the utilization of outcome and cost measurements as performance indicators. Whether concerning project teams or official units, all hospitals were engaged in some type of value-based performance measurement.

#### Outcome measurements

All hospitals we spoke to were actively involved in outcome measurements, thus trying to optimally standardize the outputs of their services—in this case, referring to the effects of these services on patients’ health. The way in which these measurements were used, however, differed from one hospital to the next. Most notably, there were differences regarding the issue of benchmarking and comparison with other providers. Some hospitals had formed collaborations in which they benchmarked their outcome data:

The approach of [hospital name] is that you benchmark the scorecard, and when you see differences, these will be discussed. And when you think one of the hospitals is doing something which leads to better results, then the others will adopt that—free of obligation, for the time being (3).

Several hospitals had been able to establish such benchmark partnerships, and those that had not seemed to recognize the potential benefits of these collaborations; some explicitly expressed the desire to form such partnerships in the future (7). Although not all hospitals were, at the time of the interviews, involved in external benchmarking, all of them were either developing or already making use of dashboards for *internal* reflection.

We are building quality dashboards, some of which are already implemented. [A]nd we use those to continuously improve the care paths, for the [multidisciplinary] teams, but we also use them for reporting to the board of directors (2).

Next to standardizing work output, value-based performance measurements seemed to have generated a boosting effect on the collaboration among team members by creating a sense of shared responsibility for particular goals:

They really start to cooperate better, being aware of each other’s problems and also solving those better with each other. [B]etween different specialties, nurses but doctors too, they will really look much better at that dashboard together: this is what we find important, this is what patients find important in terms of treatment and outcomes, and we actually think this is important too. They make dashboards that much more belong to them, which also makes them put much more effort into improvement. (7).

#### Cost measurements

With regard to value-based performance measurement, costs seemed to have received relatively little attention compared to outcomes. While all Dutch hospitals were involved in outcome measurement, several hospitals had not (yet) utilized cost measurements in their efforts to create more value-based coordination. This was exemplified by one respondent when s/he was asked whether their multidisciplinary teams were accounting for costs:

No, not yet actually. On the cost side we are struggling quite a bit to make that insightful. That is also not our focus. Our focus is: we want to improve the outcomes of care, from the philosophy that the costs will then lower automatically (9).

Among those that were measuring costs, approaches differed. Some were making use of “cost drivers”—with proxy indicators such as length of stay, without immediately connecting these indicators to hard currency (5). Several hospitals, however, had been using cost price calculations, and some had hired an external agency to make this work (11). Moreover, these cost measurements were increasingly becoming part of the dashboards that enable multidisciplinary teams to evaluate their performance.

In sum, at the time of our data collection, multiple hospitals had been struggling to gain insight into the costs of their services, yet others seemed to have made steps by incorporating cost price indicators into their performance dashboards. These differences among hospitals though, may have been related—at least partly—to the degree of official commitment from the highest levels of the organizations and the recourse allocation that comes with it.

### Training and indoctrination

For organizational redesign to be successful, whether sweepingly or incrementally, it was widely recognized that a solid support base among all levels of the organization is crucial.

Tell the story. Explain why you do this and repeat it. Repeat it. Repeat it. Repeat it. And explain, each time, this is the reason why we do this, we think changing this will work better. So, don’t begin by telling them what you are going to change, but first just start by creating the setting in which it makes sense to change (5).

To generate a deep and widespread support base within the organization, Dutch hospitals utilized particular tactics. Some applied a focused but unofficial approach, in which the executives first “look for the right informal leaders and convince them,” and then, through these informal leaders, they tried to get everyone else on board (7). But while several hospitals were handling their “indoctrination” ([17] p97-99) informally, others had officially developed internal training programs, specifically focusing on VBHC.

We are actively involved in training within those [multidisciplinary teams]. Both specifically for the daily leadership and also more broadly. [N]ext tot that, we have set up a general training program in which within the [multidisciplinary teams] they can use this training. On the one hand, that is really about clear knowledge, so “what is value-based health care, what are those [multidisciplinary teams], why do we do this, how does this match the developments in the Dutch health sector?” On the other hand, knowledge and education for a specific [multidisciplinary team] (3).

So, with regard to the provision of training and value-based “indoctrination,” some hospitals had relatively formalized frameworks in place. Others, however, found themselves making “baby steps” in developing a more coherent program (11). Additionally, there were cases in which the leadership was not actively propagating VBHC theory—even when VBHC was part of the official hospital strategy.

Really including the leadership of the hospital in that vision, that’s still missing for us. I think that this is also essential for success in the long term. [T]hat somewhere there will be a turning point from bottom-up to also top-down management. [T]hat element is still missing for us to make that switch, because that does seem like a very nice one, when you can combine that with the bottom-up enthusiasm (12).

To conclude, regarding value-based indoctrination, an important distinction we noted among the approaches of various hospitals relates to the degree of official commitment to value-based redesign, particularly from the higher levels of the organization.

### Job specialization

Value-based job specialization concerns the division of labor that is explicitly related to VBHC. Ideally, job specialization enables organizations to effectively match individual workers to their specific tasks ([17] p70-79). Within Dutch hospitals, the issue of value-based job specialization was primarily relevant regarding the leadership of multidisciplinary teams rather than the task division within those teams. This is because the actual tasks that most personnel needed to perform were usually not affected by VBHC initiatives; what did change was *with whom* people collaborated on a day-to-day basis (7).

When it comes to appointing the leadership roles within the multidisciplinary teams—the daily management referred to in the section on Unit grouping—hospitals varied in their approach. Within some hospitals, the composition of this leadership was determined organically, usually depending on the enthusiasm of particular individuals, and had thus been different from one team to the next (10). Others had clearly defined a particular set of roles for each of their multidisciplinary teams, with a clinical leader, an administrative manager, and a leading nurse, for example (1). The importance of the composition of this leadership was widely recognized, and several interviewees referred to this daily management when they were asked about a vital lesson they had learned:

For me, a big lesson is the importance of a good leader above those [multidisciplinary] teams. And this is also a challenge we’re facing. Currently, the leaders are the ones that took initiative, but they are not always the best leaders. That is something we definitely have to deal with (9).

One of the lessons is that appointing a daily leadership [group]—formally, through an application procedure—has been very beneficial (3).

In sum, some Dutch hospitals had come to develop official application procedures for the daily management of multidisciplinary teams, while others were—thus far—favoring a more organic approach.

### Formalization of behavior

In general, as a design parameter, “formalization of behavior” refers to the predetermined standardization of work processes ([17] p81-83). In health care delivery—particularly in the context of VBHC—clinical pathways for medical conditions have been a widely used form of standardization that enables a sequence of interdependent activities to be tightly coordinated beforehand.

In 2016, we started with internal VBHC clinical paths, kind of a combination of Lean and VBHC. So, really trying to streamline the processes, measuring the right outcome indicators, assessing those, and steering on that basis (8).

All of the hospitals we spoke to were involved in the development and implementation of clinical pathways around medical conditions. In many cases, however, this continued to be a work-in-progress.

### Decentralization

For the purpose of this study, value-based decentralization refers to the process by which value-based units acquire greater organizational autonomy. As with other design parameters, most Dutch hospitals had been hesitant to turn this knob. For some, it remained questionable why and to what extent such autonomy is even desirable:

In my opinion, you should first have results, in a small setting, and then see “what have we learned from this? What works and what doesn’t?” In terms of ICT [information and communications technology], dashboards, indoctrination, all those variables you take into consideration. And those, you scale up, before you start looking at structures, systems, architectures (12).

Some hospitals were starting to experiment, on small scales, with more autonomy for their value-based pilot units. In particular, this concerned financial autonomy: value-based units with their own budget control.

[T]he current integrated [value-based] units, they will start next year with sort of their own budget. You could call it a “shadow budget.” [A]nd for new integrated units we’ve set aside a kind of mandate to give them some financial leeway so that they control their own development. So, there is already something like a budget. But we are also seriously considering, thinking about, “can we really autonomize them entirely?” That’s a question we’ll be taking about (1).

A recurring theme regarding decentralization but also more generally regarding value-based redesign, was the notion of a gradual transition toward more value-based structures. Interestingly, in many cases, the final stage of this transition was not clearly envisioned but rather seen as something that would be determined later on, based on the experiences and lessons learned during that incremental process.

## Discussion

This research combines theory on value-based health care with theory on organizational structures, and explores how Dutch hospitals currently work toward value-based redesign: structural coordination around medical conditions over full cycles of care. Our study demonstrates that Mintzberg’s [17] organizational design parameters offer a useful framework to analyze the implementation of value-based health care delivery.

Interestingly, while the core literature on VBHC depicts value-based redesign as a fundamental change, with radical and sweeping implications [3, 7, 8], our study portrays a different picture: one of incremental redesign, with hospitals applying a variety of design parameters to various degrees.

The design parameter that best illustrates this contrast is unit grouping. Although one hospital did establish value-based units (through a top-down approach), most hospitals we spoke to are hesitant, at least for the time being, to (re)group into units around medical conditions. Rather, these organizations aim to spur coordination through various liaison devices, such as intermediary managers and regular multidisciplinary team meetings, leaving the original functional units intact. This contrasts with the authentic notion of integrated practice units (IPUs)—which concerns, above all, a *basis for grouping* in health care organizations [3].

Whereas our current study describes the use of liaison devices (rather than unit grouping) to enhance coordination between functional units in terms of applying VBHC principles, this can also be seen as the manifestation of a broader trend in which hospitals worldwide are trying to overlay their functional designs with multidisciplinary teams [28]. This trend within hospitals, in turn, parallels a more general tendency seen in many sectors whereby organizations increasingly become “process-oriented,” emphasizing workflow interdependencies instead of functional structures [29].

When it comes to organizational structures, VBHC coincides with the idea of process orientation, although there are some defining characteristics. Similar among both, though, is the belief that the traditional functional structures of hospitals are flawed organizational designs that can and should be overcome [2, 3, 8]. To do so, a process-based design would be built on the premise that to optimize quality and efficiency, an organization should be structured around its core business processes [2]. Accordingly, process orientation contrasts with the old and nowadays controversial dictum in organization theory that “structure follows strategy” [30]. Instead, these scholars propose that “structure follows process” [2, 29]. VBHC theory also appears to profess process orientation, but only as a consequence of seeing the specific processes (i.e. care cycles) of addressing medical conditions as *the chain of activities that generates value* for patients. Within this framework, it is first and foremost the creation of value that should determine organizational structures [8]. Hence, if there were a VBHC variant of the old dictum, it might be something like “structure follows process follows value creation,” or maybe just “structure follows value.”

Whatever the phrase, the point would be that health care organizations should structurally coordinate their work activities such that value for patients is optimized. And hard-line adherents of VBHC are convinced that this requires a radical transformation toward IPUs for medical conditions, rather than just overlaying a (dys)functional structure [3, 8].

However, an important finding of our study, one that mirrors accounts on process orientation [31], is that the prevailing existence of a functional structure does not imply a complete absence of value-based redesign. Indeed, our study demonstrates that although the more radical switch to value-based units remains rare in the Netherlands, this does not preclude other forms of value-based redesign from taking hold. For example, aside from the aforementioned liaison devices, hospitals utilize planning and control systems (i.e. outcomes and costs measurement) to upgrade coordination around medical conditions. Scorecards and dashboards containing outcome measurements are universally used for internal evaluation, but not all hospitals participate in benchmarking with other organizations. And although the use of cost measurements is less prevalent, several hospitals conduct cost accounting and relate this to outcome data. None of the hospitals we spoke to, however, actually measures or estimates patient costs over full cycles of care.

Overall, Dutch hospitals aim for *incremental redesign*. These organizations employ a variety of design parameters to various degrees. They generally envision a transition toward *more* value-based structures, but this is usually described as a “slow process,” starting with “experiments” and “pilots,” characterized by “baby steps.” Moreover, the envisioned end point of this transition—the quintessential design—remains unclear; the idea is that this will be determined along the way. So, even when the core principles of VBHC are widely embraced, many of our interviewees do not acknowledge IPUs as the pinnacle of structure in health care.

For several reasons, an incremental approach to value-based redesign may well be more viable than the radical transformations professed by Porter [3, 8]. To start, organizations generally tend to hang on to their structures for relatively long periods of time [17, 30], and this appears to apply to hospitals as well. Additionally, profoundly changing well-established behavioral patterns is often resisted [17], and studies have well documented such resistance within health care organizations [28]. This may at least partially explain why, in reality, most organizational restructuring does not occur radically but rather incrementally, through continuous modifications of existing structures ([17] p105). Moreover, the long history of the functional design of hospitals has left deep imprints on work practices, professional identities, and social norms within these organizations [28]. This type of historical impact is not easily swept away, and creating multidisciplinary *units* in hospitals by itself is *not* enough to overcome the extensive reliance on disciplinary boundaries in everyday health care delivery practices [28]. Breaking down these “invisible walls” will require additional time and effort [28]. Therefore, an incremental approach to value-based redesign (rather than a radical one) seems better suited to do justice to the history of medicine (rather than sweeping it away), while also allowing interdisciplinary collaboration to evolve over time (rather than enforcing it immediately), and thus appears (as far as we can tell) a more viable avenue for the adoption of VBHC principles than the (fundamentalistic) one professed by their originators [3, 7, 8].

This study has at least four important limitations. First, it should be noted that while Mintzberg’s design parameters have proven to be highly useful for analyzing value-based redesign, our use of this framework has undoubtedly shaped our findings. Second, our study focuses on Dutch hospitals; the existing policies, regulations and financial arrangements in the Dutch health sector have likely molded how these organizations may or may not pursue a more value-based design. Third, we interviewed only one representative per hospital, most of whom did not have a clinical role. Interviewing only one (nonclinical) representative per hospital may have generated biased pictures of what is happening within the individual hospitals, which could potentially have spilled over to our aggregate findings. Fourth, while our conceptualization of “value-based design parameters” may be useful for analyzing and implementing VBHC, it was beyond the scope of this study to examine the *effects* of utilizing these design parameters on performance. We strongly encourage future research regarding the results of value-based redesign.

### Practical implications

Hospital executives, managers and leading physicians who want to upgrade coordination around medical conditions have a variety of organizational knobs to turn to various degrees. Our study indicates that many providers will likely favor incremental redesign over radical transformation. Considering hospital units, rather than radically regrouping the entire organization into units around medical conditions, a more incremental approach could be one in which a hospital first experiments with one or a few condition-based pilot units (around breast cancer or maternity care, for example). Depending on how the pilot proceeds, modifications can be made, such as granting more financial autonomy to the respective unit.

If, at least for the time being, (most) traditional specialty units are left intact, coordination around medical conditions can still be enhanced in various ways. For instance, hospitals could appoint one or more (value-based health care) managers, whose roles are first and foremost to foster interunit communication and coordination. A common way to do this is by forming multidisciplinary teams around a medical condition, with members from various specialty units meeting on a regular basis. One point of discussion during these meetings can be how to improve value for patients with a similar medical condition—based on value-based performance measurements (e.g. outcomes and costs).

It is widely recognized that structural changes, whether sweepingly or incrementally, benefit from a solid support base across all levels of the organization. Concerning value-based redesign in Dutch hospitals, systematically propagating information (through training programs, for instance) has been regarded as a useful way to generate awareness and support throughout the organization.

Multiple Dutch hospitals initially struggled with the composition of the leadership of their multidisciplinary teams. Their experiences indicate that the characteristics of these leaders matter: it is probably good to have multiple leaders, each representing a particular organizational component (e.g. administrative, nursing, business intelligence), and several hospitals have come to favor official application procedures over automatically granting leadership to the most enthusiastic physicians.

Ideally, hospitals would not have to repeatedly develop all of these approaches by themselves. Instead, the path toward more value-based structures could be built on the efforts and lessons of others. Therefore, we encourage providers to gather information, evaluate proceedings and report on their experiences; this can give rise to a knowledge base on which value-based redesign may be founded.

## Conclusions

Value-based redesign is not necessarily a matter of radical changes or binary choices between traditional structures on one side and value-based designs on the other. Instead, inspired by the idea to achieve the best outcomes as efficiently as possible, hospitals are *incrementally exploring various ways* to improve coordination around medical conditions over full care cycles. Our study demonstrates that Mintzberg’s [17] organizational design parameters offer a useful framework to analyze the implementation of value-based health care delivery. Hopefully, our conceptualization of “value-based design parameters” offers guidance to providers who find themselves in search of more value-based structures. Moreover, we hope the framework we sketched here can assist research on and the evaluation of what works—e.g. which knobs might be turned, to what degree, in which contexts—in terms of value for patients.

## Supplementary Information

**Additional file 1. **Theory on design parameters.

**Additional file 2.** Interview guide and topic list.

**Additional file 3. **Coding scheme.

## Data Availability

The dataset analyzed during the current study is available from the corresponding author on reasonable request.

## Abbreviations

VBHC: Value-based health care
IPU: Integrated practice unit
