# Supplementary material for: Redesigning value-based hospital structures: a qualitative study on value-based health care in the Netherlands
Source: BMC Health Serv Res. 2022 Sep 22;22:1193. doi: 10.1186/s12913-022-08564-4 (PMC9502905; doi:10.1186/s12913-022-08564-4)
Supplement: Supplementary file 1 — Additional file 1. Theory on design parameters. [file 12913_2022_8564_MOESM1_ESM.pdf]

Additional file 1. Theory on design parameters

Mintzberg states that all organized work requires (1) a certain division of labor into tasks, and (2) the coordination of those tasks. And, roughly speaking, an organizational structure is determined by the ways in which these two requirements are addressed [1(p2)]. He goes on to distinguish five basic **coordinating mechanisms** [1(p3-6)]: five general ways by which work can be coordinated:

- First, there is *mutual adjustment*: coordination through informal communication.
- Second, coordination via *direct supervision*: someone taking responsibility for and overseeing the work of others.

The third, fourth and fifth coordinating mechanisms all have to do with the *standardization* of work.

- The third mechanism concerns the *standardization of work processes*, whereby the actual content of the work is preprogrammed (usually through protocols or fixed instructions).
- Fourth, there is the *standardization of outputs*, whereby the results of the work (the outcomes of processes) are planned and monitored.
- Fifth, there is the *standardization of skills*, whereby the workers themselves (physicians for example) are standardized, usually through specified training.

The structuring of organizations then, refers to "turning those knobs" that affect the division of labor and modify the mechanisms that coordinate work within an organization [1(p65)]. In slightly more technical terms, those knobs are described as the "*design parameters*" of organizational structures. Mintzberg distinguishes eight design parameters. Below we will briefly discuss each of them, while also highlighting how each knob relates to value-based redesign (within the framework of our study).

From Mintzberg's knobs to "value-based design parameters"

1. Unit size: the number of employees that are grouped together can affect the way organizations coordinate their work in various ways [1(p134-146)]. Within this study, we will focus on considerations regarding unit size that are explicitly related to the principles of VBHC.

2. Unit grouping: fundamentally, this design parameter builds an organization's structure of *formal authority*. Through grouping, work is coordinated by establishing direct supervision, and additionally, by encouraging mutual adjustment among team members. Mintzberg distinguishes two opposite bases for grouping: market-based and functional grouping. In market-based grouping, organizations compose their units based on the *ends* of work processes (i.e. characteristics of the markets they serve). By contrast, functional grouping is based on the *means* of performance – the *functions* (including knowledge and skills) that are used to create a product or service [1(p104-14)]. Traditionally, most hospital units are grouped by function (e.g. specific knowledge and skills) rather than markets (e.g. particular patient characteristics). Considering VBHC however, unit grouping would primarily be based on the *ends*: patients with a certain medical condition. Thus, within this study, *value-based grouping* refers to the establishment of hospital units around medical conditions.

3. Liaison devices: these types of knobs are designed to override the limitations of particular forms of unit grouping. In principle, they constitute *contacts*, such as meetings and posts/positions, that are overlaying the formal structure to facilitate mutual adjustment. So, whereas turning the unit-grouping knob encourages mutual adjustment *within* units, liaison devices are used to attune coordination *between* units [1(p161-77)]. Within this study, we will focus on formally established contacts between units that are aimed at coordination around a medical condition (over the full cycle of care).

4. Planning and control systems: turning this knob will primarily enable coordination through the standardization of work *outputs*. Plans can specify output standards; control systems serve to measure whether these standards are met. At its core, planning and control systems have two main objectives: to monitor and to motivate [1(p148-51)]. As mentioned, within VBHC the overarching goal is value optimization, with value defined as health outcomes over costs [2]. Thus, within this study, outcomes and costs constitute key performance metrics, and *value-based planning and control* will refer to such measurements, plus the way in which these are used to motivate teams.

5. Training and indoctrination: turning this knob enables an organization to standardize its workers' skills (training) and norms (indoctrination). In this study, we will focus on indoctrination, since most of the work in hospitals is conducted by medical professionals who have acquired their main skillset through previous education. Organizational indoctrination is aimed at establishing certain norms, and to ensure that decision-making occurs in the interest of the organization [1(p95-103)]. In this study, we focus on the explicit proliferation of VBHC-theory as being *value-based indoctrination.*

6. Job specialization: turning this knob is the key manner by which division of labor occurs. Ideally, job specialization enables organizations to effectively match individual workers to their specific tasks [1(p70-9)]. So, what would/could "value-based" job specialization look like in hospitals? In this study, we think of such specialization as the *division of labor* that is explicitly related to VBHC. For instance, appointing a VBHC-project leader, or having a certain portion of someone's time dedicated to a VBHC-project.

7. Formalization of behavior: turning this knob concerns the standardization of *work processes*. Thus, this design parameter coordinates various activities by *regulating the actual content of work*. Through formalization of behavior, an organization can tightly coordinate

activities *in advance*. This often takes place through protocols and other formal regulations (the musical notes given to an orchestra, for example) [1(p 83-4)]. Regarding VBHC, this study focusses on formalization of behavior aimed at the coordination of healthcare delivery *around a medical condition* and over the *full cycle of care*.

8. (De)centralization: this parameter concerns the *design of decision-making processes* within an organization. This knob can be turned both ways: to centralize is to consolidate decision-making power; decentralization concerns the distribution of such power [1(p182-84)]. Within this study, *value-based decentralization* will refer to the distribution of decision-making power to value-based units.

References

1. Mintzberg H. The structuring of organizations. Englewood Cliffs, N.J.: Prentice-Hall; 1979.

2. Porter M. What is value in health care. N Engl J Med. 2010;363:2477–81. https://doi.org/10.1056/NEJMp1011024.
