# Supplementary material for: Redesigning value-based hospital structures: a qualitative study on value-based health care in the Netherlands
Source: BMC Health Serv Res. 2022 Sep 22;22:1193. doi: 10.1186/s12913-022-08564-4 (PMC9502905; doi:10.1186/s12913-022-08564-4)
Supplement: Supplementary file 2 — Additional file 2. Interview guide and topic list. [file 12913_2022_8564_MOESM2_ESM.pdf]

Additional file 2. Interview guide and topic lists (for interviews and focus group)

Interview guide

Introduction

Background and research objective; informed consent and anonymization.

**Main questions**	**Topics**
What is your role within the hospital?	
How is your hospital working on coordination and organization around medical conditions?	Organizational structures, multidisciplinary teams
To what extent did the hospital shift (i.e. change) its (traditional) organizational structure towards units for medical conditions?	Unit grouping, liaison (matrix)
To what extent do your units/teams have formal authority?<br>How and to what extent are your units/teams held accountable for their performance (outcomes/costs)?<br>How are decisions made about scarce resources?	Unit grouping, decentralization, authority, accountability, decision-making power
What is the composition of your multidisciplinary teams/units (who is in it, how many, and why)?	Unit size, unit grouping, liaisons, job specialization
How is (informal) contact and coordination of work/tasks facilitated within teams / between unit members?<br>How is (informal) contact and coordination facilitated across units/teams?	Liaison (positions, committees), formalization of behavior
To what extent are tasks and responsibilities formalized within teams/units?	Formalization of behavior
What does the planning and control cycle of MD teams / condition-based units look like?	Planning and control, outcomes, costs
How are outcome measurements monitored?	Outcome measurement, liaison

How are costs measured and monitored?	Cost accounting
How and to what extent do you proliferate knowledge, information, norms and skills about VBHC and coordination around medical conditions?	Training and indoctrination
What have you learned thus far, about what works and what does not?<br><br>What do you see as challenges for proceeding with the condition-based organization of health care?	
**Additional questions**	
What is the minimal size of a unit/team?	Unit size
What does the leadership of a unit/team look like? (How are teams/units guided?)	Liaison, job specialization
How is (informal) contact and coordination facilitated across units/teams?	Liaison
To what extent, how often, and on which topics do meetings take place among team members? And meeting between the unit/team and other layers in the organization (such as the board)?	Liaison
Do units/teams have and/or make their own annual budget?	Planning & control,<br><br>decentralization
How do multidisciplinary teams come into being within your hospital?	

Topic list focus group

- The progressions of Dutch hospitals regarding organization around medical conditions (VBHC).
- What may we have overlooked (i.e. hospitals, teams, design parameters)?
- Organizational redesign: completely switching vs. matrix structure.
- Measuring costs (challenges).
- Top-down versus bottom-up creation of multidisciplinary teams.
