# Supplementary material for: Redesigning value-based hospital structures: a qualitative study on value-based health care in the Netherlands
Source: BMC Health Serv Res. 2022 Sep 22;22:1193. doi: 10.1186/s12913-022-08564-4 (PMC9502905; doi:10.1186/s12913-022-08564-4)
Supplement: Supplementary file 3 — Additional file 3. Coding scheme. [file 12913_2022_8564_MOESM3_ESM.pdf]

Additional file 3. Coding scheme (value-based hospital structures)

Main codes	subcodes
Initial (deductive) codes	
• DP Unit size	
• DP Unit grouping	- Market-based (grouping)
• DP Liaison devices	- Commission (liaison)<br>- Position (liaison)<br>- Matrix (liaison)
• DP Planning & control systems	- Outcomes<br>- Costs
• DP Training and indoctrination	
• DP Job specialization	
• DP Formalization of behavior	
• DP Decentralization	
Additional (inductive) codes	
• Transition	
• Interdependencies of work(flow)	
• Strategy/vision	
• Extra/not specified	
